# Flowing data: women’s views and experiences on privacy and data security when using menstrual cycle tracking apps

**DOI:** 10.1093/oodh/oqaf011

**Published:** 2025-05-17

**Authors:** Sarika Mohan, Judy Jenkins

**Affiliations:** Health Data Science, Swansea University Medical School, Swansea University, Health Data Science Building, Office Number 102 First Floor, Singleton Park, Swansea SA2 8PP, UK; Health Data Science, Swansea University Medical School, Swansea University, Health Data Science Building, Office Number 102 First Floor, Singleton Park, Swansea SA2 8PP, UK

**Keywords:** period tracking apps, data privacy, information security, fem-tech, user experience, personal informatics

## Abstract

Menstrual cycle tracking apps are mobile applications that help female users track their menstrual cycle and gain future period predictions. Although these apps have advantages, they have been criticized for their lack of accuracy in prediction and poor adherence to privacy laws. This qualitative study aimed to explore the experiences and perceptions of users of period tracking apps with a focus on data security and privacy. Twenty-five female users between 19 and 38 years of age who have experience with menstrual app usage were interviewed via online video conferencing tools using a semi-structured interview technique. Data analysis was done using inductive thematic analysis, and eight themes were identified. The participants stated that they prefer apps that provide good period predictions and have a better user interface. They also expressed a desire to have ownership over their data and their access and that the apps should provide clearer privacy statements. The results of this study are vital for app developers to consider when designing or updating their apps to ensure that it is suited for a diverse group of end users. They must also implement stricter data protection measures so users can trust the apps with their information. Further research needs to be conducted to gain insights from different cohorts of users.

## Graphical Abstract

**Graphical Abstract:**
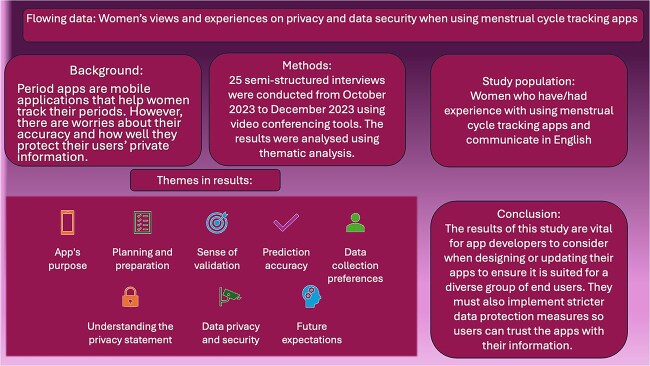

## INTRODUCTION

Person-generated health data (PGHD) refers to health data that individuals generate outside the traditional healthcare facility [1]. These data are also used to longitudinally track and manage specific medical conditions or concerns [1]. The fem-tech industry, which stands for female technology, is a fast-growing industry focused on improving female health and well-being [2]. Fem-tech applications encompass a wide range of solutions, from mobile applications to medical devices in both the public and private sectors [3]. Menstrual cycle tracking apps are mobile applications in the personal informatics section of fem-tech that are designed to help menstruators track their menstrual cycle and receive predictions for future cycle dates [4]. These tracking apps have dominated the fem-tech industry since they were first introduced and have gained popularity rapidly in the last 5 years [5]. On one hand, studies indicate that the integration of consumer health informatics and social media in tools such as period tracking apps can be valuable in identifying and addressing the obstacles and negative perceptions associated with accessing healthcare [6, 7]. On the other hand, these apps have also been criticized as being designed to cater to a specific group of female population [3, 8].

## BACKGROUND AND RATIONALE

Earlier qualitative studies that were conducted to understand female users’ perspectives on using apps for tracking menstruation show that these apps have improved the users’ knowledge about their menstrual cycle and made them more aware of their reproductive health [9, 10]. Few studies were conducted solely to understand the purpose of using fertility and menstrual tracking apps [11, 12]. Saugar *et al*. [13] conducted a scoping review to elaborate on the factors used by period apps to improve their prediction accuracy. Moglia *et al*. [14] evaluated the prediction accuracy of these apps and listed the apps according to their performance and gave recommendations as well. Apart from users’ self-awareness, the data collected by these self-tracking apps provide researchers with a fresh source of information that was previously inaccessible and difficult to obtain [15]. Therefore, recent studies have been conducted to gain a better understanding of menstruation using anonymized data from the app’s database [16, 17]. However, critics question the privacy, ethics and data security policies of these menstrual apps because of the extensive amount of personal and sensitive data that are collected and handled without transparency [18]. Baltzer and Bonacina [19] studied the privacy policies of different menstrual apps and revealed that most of the apps show poor adherence to General Data Protection Regulation (GDPR) regulations. Past studies conducted on women’s use of period apps and their willingness to share period data with their partners have failed to examine their perspectives on data privacy [20, 21].

After conducting an extensive literature review on menstrual apps and their ethics, a few research gaps were discovered with the most prominent gap being a lack of focus on recording the end users’ perspective. Therefore, this qualitative study aims to explore the experiences and opinions of women using menstrual cycle tracking apps with a special focus on privacy and security of the data that are regularly collected by them. The objectives of the study are to comprehend why women opt for certain tracking applications over others and to record their perception of how it contributes to the overall improvement of their health. Additionally, it seeks to explore women’s awareness of the privacy policies implemented by period tracking mobile applications.

## MATERIALS AND METHODS

The aim of this study was to understand the previously unexplored views on privacy by women who have used menstrual cycle tracking apps, so a qualitative approach was chosen [22]. Individual semi-structured interviews were chosen as the data collection tool because of their flexibility and versatility in form [23]. The interview questions were chosen with caution, by referring to other studies, as the topic of discussion was sensitive, and were double-checked to not make the participants feel uncomfortable and to avoid interviewer bias [24]. The purposive sampling technique was used as it was an essential characteristic to interview individuals who have used a menstrual app [22]. The inclusion criteria for participation were users or past users of menstrual tracking apps, who were older than 18 years of age and are able to communicate and express their thoughts clearly in the English language. It was necessary to include participants who have stopped using the apps as well, to understand their reasons. In order to include participants from different regions, all interviews were held online using Microsoft Teams and Zoom video conferencing tools. No compensation was provided to the participants for taking part in this study.

### Data collection

Ethical approval was obtained from the Swansea University Medicine ethics committee [Reference number: 22023 7807 6894], and the study was conducted in accordance with the ethical standards of the Helsinki Declaration. The study was advertised from October 2023, on multiple social media platforms [Instagram, Facebook and LinkedIn] using a newly created public account with the name ‘period_apps_study’. A digital poster was shared containing details about the aim of the study, the inclusion criteria and contact details of the principal researcher. The data collection period was from October 2023 to December 2023. Interested individuals who emailed the researcher were all sent a copy of the participant information sheet and further details about the study. Those who agreed to participate and fit the inclusion criteria were selected and scheduled for interviews at a suitable time. All participants filled the consent form online using Microsoft Forms, and the completed forms were stored in a separate password-protected folder. All the interviews were conducted by a single researcher. The interview questions are listed in the appendix (Appendix A: Semi-structured Interview Questions). The complete interview was video-recorded and auto-transcribed by the video conferencing tool, and the transcripts were cross-verified for accuracy. The interview ranged from 30 to 45 minutes in duration. The interview questions acted as an initial guide for the participants, and further questions were asked based on their answers. Data saturation was reached with 25 participants, as observed in other studies [25, 26].

### Data analysis

Inductive thematic analysis was chosen as the data analysis method since the goal of this study was to solely understand the participants’ views without any pre-existing theories or assumptions [27]. Braun and Clarke’s six-step process to thematic analysis was followed [28, 29]. After assigning pseudonyms for the participants, the data were familiarized by rereading it. Initial codes were assigned using Microsoft Excel, and different themes emerged by grouping these codes. The themes were reviewed again to ensure that the data fit the themes. The final eight themes were chosen to ensure that they accurately reflected the views of the participants, in a coherent way, and the quotes from participants that were most suited for the theme were also added [27].

## RESULTS

The participants ranged in age from 19 to 38 years and were from different parts of the world. The distribution of app usage and the country of residence of the participants can be found in Table 1**,** and the age and education levels can be found in Table 2. The most popular apps used by the participants were the health app by Apple, Clue and Flo. None of the participants used paid apps or any paid services.

**Table 1:** App usage and country of residence

**Menstrual app used (in italics) and country of Residence**	**Count**
***Apple Health app***	***6***
Canada	1
India	1
Jordan	1
Malaysia	1
UK	2
***Clue***	***7***
India	3
Jordan	1
Singapore	1
UK	2
***Flo***	***6***
India	3
Nigeria	1
UK	1
USA	1
***My calendar***	***3***
Malaysia	1
Nigeria	1
UK	1
***My period***	***2***
Germany	1
India	1
***Period calendar***	***1***
China	1

**Table 2:** Age and education level

**Education level**	**18–21 years**	**22–25 years**	**26–30 years**	**31–40 years**
Bachelor’s	4	6	4	0
Master’s	0	6	3	2
Total	4	12	7	2

### App’s purpose

Almost all participants said that the primary reason for using a period tracking app was to monitor and gain insights into their bodies. Other reasons included tracking their irregular periods in general or due to medical conditions such as polycystic ovarian syndrome (PCOS) or other systemic conditions. Exporting their data as a document and sharing it with doctors was also a helpful feature. Few switched from the conventional pen and paper technique to using an app due to convenience. *‘It is easier to just log in my periods on an app rather than actually physically writing them down’* [Participant-1].

The app’s interface, design, user reviews and peer recommendations greatly influenced participants’ choice of app. *‘All the information is in a single page, so I don’t have to like keep clicking and hovering over pages to understand’* [Participant-20]. Few participants further explained how they used other apps that were not suited for them, or their family member, so they switched to their current one. In addition, apps that provide functionality without a subscription fee and back up their data to prevent data loss when switching devices were a few other reasons.

### Planning and preparation

Many participants mentioned that the app’s notification feature was the most useful feature, as it alerts them about an upcoming period and they prepare themselves by carrying sanitary products, medicines or by wearing pantyliners. *‘My blood pressure goes down very low, so I have to be vigilant on my first day and I have extreme abdominal pain, cramps and everything…and I have to take my ORS [Oral rehydration solution] to make my blood pressure normal’* [Participant-12].

Participants also plan their travel, diet and workload according to the prediction. *‘I also try to avoid any strenuous work or religious occasions during that time’* [Participant10].

Certain participants use the app’s prediction of the fertile period to plan their sexual activity based on their intentions with conception. Participant-21 mentioned switching to a different app after they became pregnant as it provided better nutritional guidance throughout their pregnancy.

### Sense of validation

Period tracking apps help in recording the characteristics of their period such as flow; colour; clotting; spotting; and other menstrual symptoms such as mood swings, cramps, bloating, tender breasts, intensity of abdominal pain and migraines. Participants find it beneficial for self-care and helped them reflect on their day and learn more about their own bodies.

Some participants have linked stress, mood swings and increased libido to different phases of their menstrual cycle as these symptoms were consistently present during that phase. Therefore, it gave them a sense of validation and reassurance that these feelings were due to their menstrual cycle, and it also helped a few to try and overcome them. *‘I am feeling this way because of my hormones, so I become mindful when I get negative thoughts and ignore it’* [Participant-8].

While not all participants believe that recording their subjective symptoms improves prediction accuracy, many still appreciate accessing more information and reducing stigma around periods and reproductive health conditions. *‘The stigma around PCOS has reduced and not treated like a terminal illness!’* [Participant-22].

### Prediction accuracy

Opinions on the accuracy of the app’s period prediction were almost equally split, with some participants finding it extremely accurate and others not. *‘It is almost always accurate’* [Participant-16]. On one hand, the participants who praised the apps for its accuracy mentioned that it helped them plan their days effectively. On the other hand, participants who expressed dissatisfaction with the accuracy said that they did not trust the app’s predictions and had to track their period separately, due to the app not accommodating for different cycle lengths.*‘ It’s only useful when you are on a regular [cycle] like 28 days cycle or 31 days cycle’* [Participant-3].

Moreover, users elaborated further by saying that it created feelings of anxiety and added to the stress as they prepare themselves for an upcoming period unnecessarily and forced them to question the quality of their health. *‘I feel that it that makes me very anxious because I’m not getting my period and might be pregnant’* [Participant-5]. Participant-24 said she stopped tracking her symptoms because *‘I also felt that my mood or the pain level changes according to the predictions.’.*

Five participants reported that they switched to a different app or stopped using apps altogether, opting for a physical calendar due to the poor predictions of their previous app.

### Preferences with data collection

Despite the provision to input various types of data into the app, many participants prefer to only track their menstrual cycle. They also commented on the amount of data being collected by the period tracking apps as being excessive and not relevant to their menstrual cycle. Participant-6 said, *‘Maybe when I’m on my period, I don’t even feel like I want to do it, you know, I just feel like it’s tedious.’* Many participants complained that recording additional factors did not influence the app’s period prediction. Three of them stated that they stopped using the period tracking apps because they felt uncomfortable with the amount of data collected by the app. Participant-2 said, *‘They generally ask for [hesitates] like sexual activity and things. I’m not sexually active and I don’t prefer these questions.’* Among the sexually active app users, only two felt comfortable recording their sexual activity on the app. The majority of the participants said that although it might be useful to track sexual activity, cultural and personal reasons prevent them from doing so.

Moreover, the country’s views on women’s reproductive rights influence the amount of information users are willing to enter into the app. Participant-10 said, *‘I am privileged enough to use an app to store my data and I don’t have to receive any taboo or stigma over it or my reproductive health or have anti-abortion laws’,* while Participant-19 said that they would never record their sexual activity due to fear of criminalization *‘because what if I have, like, an unplanned pregnancy and I decide to get an abortion and it’s not really legalized’.*

### Understanding the privacy policy

Most participants admitted to not reading the app’s terms and conditions of the app or privacy policy thoroughly. Participant-20 said that they *‘prefer just skipping the entire thing and then just accept it’.* Participant-23 mentioned that they found the text difficult to understand due to overly technical language, saying, *‘at some point the terms become too jargon based and you’re like, what’s happening here?’.*

Moreover, all the participants unanimously agreed that they would read and understand the privacy policies if it was simple and easy to comprehend. Participant-25, who read the entire privacy policy of their app, found a lack of clear instructions for limiting external access to their data, despite it being mentioned in the statement. They also suggested, *‘If they can summarize… just use better graphics or like you know if you have let’s say a table saying, you know this is the data we collect, this is what we do with it’.* Participant-16 noted that the consent for the privacy statement has not been renewed in the 5 years of her app usage, despite changes in the privacy policies.

### Data privacy and security

Many participants expressed scepticism towards the security of their data when asked about their thoughts on data security in period tracking apps. They admitted that they do not trust these apps with their personal and sensitive information, fearing that their data might be hacked or leaked, and therefore, practice data-safe measures to the best of their knowledge. Some participants prioritize data security by not storing their data in the app’s cloud storage and opting for local storage on their devices.

Participant-4 stated, *‘I became more cautious after moving to the U.S., as I felt that information privacy is not something that is majorly respected’.* Therefore, they switched to using their period tracking app in ‘Incognito mode’, to avoid sharing personal identifiable information. They also acknowledged that this greatly limited the usage of the app’s features that they are willing to forego for the purpose of privacy. However, despite these measures, they expressed frustration and noted that they received targeted advertisements on their social media accounts for period products during their period. Participant-25 mentioned a lack of trust in the app’s data security promises, yet continues due to the lack of alternatives. They also revealed that *‘Every time I get my periods, then whenever I used to open like Instagram or Facebook somehow, I used to get all advertisements related to tampons and sanitary napkins!’.*

Several participants considered uninstalling their period tracking apps and stopped recommending them, especially after a specific menstrual app was found guilty of selling their users’ data without their knowledge. Participant-17 particularly noted that *‘there was a wave of people saying stop using period tracking apps and I thought about it, but then all these things happening… I think it was because of Roe v. Wade’.* Participants from countries with restrictive abortion laws stated that they feel the need to take extra precautions when using these apps. Participant-19 divulged that *‘most people are scared rather than the app itself, but the fact that this the government has access to this information’.*

### Future expectations

Many participants expressed that they want complete control and ownership over their data, including who has access to said data produced by the app. Beyond that, the ability to choose the information that can be stored in cloud storage, data encryption and password or fingerprint protection for accessing the app were some of the data security measures suggested by them.

Regarding data confidentiality, Participant-7 stated that ‘*the laws that apply for doctor and patient relation should apply for this as well’*. Consent for selling their anonymized data and knowing where the data are being sold to, along with the purpose of using it, was the key statement that was affirmed by a lot of participants. Participant-16 said, *‘I just want to be aware before my data is being sold and why it is being sold’.* Numerous participants confirmed that they would like to receive a notification if their data are used and want to know the purpose of their data usage, to make sure it aligns with their values and beliefs.

## DISCUSSION

The main purpose of using menstrual cycle tracking apps is monitoring and tracking the users’ menstrual cycle, which is similar to the results observed by previous studies as well [21]. As concluded by Josephy *et al*. [30], participants agreed that apps helped them record a wide variety of period characteristics and symptoms, when compared to the traditional pen and paper technique. The ability of the app to be receptive of their users’ behaviour and the optimum time for notification delivery has been associated with increased app engagement as well [31]. The accuracy of predicting menstrual cycles in these period apps was found to be one of the factors influencing users’ preferences for a specific app [32]. As emphasized by Ammenwerth [33], the Technology Acceptance Model and Unified Theory of Acceptance and Use of Technology can be used to explain some aspects of the users’ behaviour with health information technology where perceived ease of usefulness played a significant role in continued usage of the app.

It is interesting that although many participants have reported irregular periods, the app’s prediction does not adapt and can urge the user to log their period when it has not arrived. It is therefore necessary to train app algorithms with non-standard cycles, to avoid cycles longer than 28 days to be classified as inaccurate or skipped tracking by the app’s algorithm [34]. This can also help normalize variations in the menstrual cycle due to physiological reasons and reduce the user’s stress [35].

Additionally, it is worth noting that women prefer having personalized and customized options and this study has pointed out the limited diversity in the design of menstrual apps, specifically in terms of their target audience. Despite previous research indicating the influence of lifestyle habits and ethnicity in the menstrual cycle, these apps do not provide provisions for accounting for these factors and adjusting their predictions accordingly [36, 37]. Kim and Park [38] state that according to the Health Information Technology Acceptance Model, users’ app engagement depends on its relevance. This is consistent with why certain users discontinue and switch to an app more suited to their needs.

It was evident that many users felt that period tracking apps collect more data than necessary. Studies also support this statement and show that these apps collect and store more than essential personal identifiable information, device data and health data [39]. Therefore, although research indicates that period apps can effectively predict the fertility window, this study shows that there is some hesitancy among women in utilizing it [40]. The concerns stem from the perception that a higher number of factors need to be measured to track their fertile window, and yet, this excess volume of data required does not necessarily enhance their prediction accuracy. The findings in terms of data vigilance are also in contrast to the results reported by Anderson *et al*. [25], as participants are more concerned about data leaks and their privacy in this context. Two participants emphasized their dissatisfaction about having period product advertisements on their social media, which aligns with the critique by Amelang [41] of app creators, for improvising algorithms to better process their users’ data for commercial use, rather than to improve their prediction accuracy.

This heightened attention to monitoring their data among app users can be attributed to the recent developments in women’s reproductive rights, more specifically, the withdrawal of abortion as a constitutional right in the USA [42]. Multiple Non-governmental Organisations (NGOs), legal scholars and women’s organizations urged users all over the world to reconsider using menstrual cycle tracking apps [43, 44]. Moreover, the recent analysis of privacy policies of multiple period tracking apps has revealed that these apps profit from selling their users’ data without the knowledge or explicit consent from the respective users [41, 45, 46]. Furthermore, it was widely publicized that period tracking apps have been found guilty by law, of collecting and sharing personal data with third parties without the users’ awareness [47]. Additionally, the lack of uniform privacy laws across different countries and regions, along with the ambiguity in classifying the app’s data as sensitive health data, has raised concerns [48, 49].

However, despite these drawbacks and complaints, users in this study and others are willing to continue to use these apps [50]. Barth and de Jong [51] state that this contrasting behaviour can be explained by the privacy paradox phenomenon, where users value the technology’s popularity and usability over the risk of personal data abuse. Privacy paradox is when there is a stark contrast between the privacy concern and the consumer behaviour, where they disclose sensitive information [52]. The participants in this study could be considered as mostly privacy pragmatists with a small proportion of privacy fundamentalists [53]. Although there is lack of evidence to explain the privacy paradox, the difference between the behaviour and attitude of the participants in terms of sharing data can be partially explained by various models such as the risk and trust model and the privacy calculus theory [54]. Meier and Krämer [55] state that when the privacy risk is disclosed completely, the individual’s decision to disclose their private information depends on their analysis of the benefits of it. The participants mentioned that they use a few data security measures such as entering non-identifiable information, using local storage and preferring incognito mode. However, Siapka and Biasin [39] criticize the expectations that these apps have put on their users to self-manage their privacy. They postulated that this behaviour cannot be expected from everyone and can result in consent fatigue. Thus, it is essential for the app developers to produce well-engineered/well-developed apps with stronger privacy protection aspects in order for the users to forego their inhibitions when it comes to privacy protection. [56, 57].

The lack of readability of privacy policies is common across most health apps and is another source of worry [58]. Users expressed that their biggest fears were their data being processed by unknown third parties and for irrelevant reasons [59]. This concern about data sharing is amplified by menstrual data, as women have long been taught to keep their menstruation experiences private and avoid open conversations [60]. Users who have tried to read the complete privacy statement out of their personal interest state that the statements seem difficult and intentionally confusing and that there is no clear distinction between opting out of data collection and not using the app entirely [61]. Therefore, critics raise concerns about the purpose of menstrual cycle tracking apps that lack a medical background, gather irrelevant data, have incomplete privacy policies and use marketing trackers, as being a front for data-selling warehouses [18].

### Recommendations

Improvements in the fem-tech industry are essential for the overall improvement of society, because the end users are usually responsible for their personal health and the well-being of their families [62]. Starting from the app design, there is a need to incorporate female-centred app developers, who have an understanding of the needs of their diverse end users [63]. The participants emphasized the importance of customizing the app’s content to align with the users’ goals, as it helps to create a more comfortable user experience [64]. Data security has become a top priority rather than being optional for most users, and the security measures suggested by the app users must also be taken into consideration. Therefore, app developers must try to incorporate end users in their development process to create apps that truly address their needs and worries [65]. Moreover, although few apps have the features that users require, due to a lack of awareness, they do not choose them. Therefore, it is also important to implement ethical marketing strategies to inform users about the efficiency and quality of their apps [66].

An interesting finding in this study is that women are intimidated not by the app, but by external parties accessing their data [44]. Moreover, data ownership and distribution have become complex, as multiple parties have access to the data and it has become relatively easy to re-identify previously de-identified data [67]. Therefore, the users expect more stringent laws to govern these period apps, with the highest level of confidentiality to be applied to their data [68]. In addition, to avoid the fear of criminalization, users should be in charge of access control for their personal data by using privacy dashboards, where consent can be given or withdrawn, or have their personal data not be recorded completely [69]. Regarding menstrual research, users are willing to share their data, provided consent is asked to obtain their data in an anonymized format and the researchers clearly explain the purpose of their study [70]. These recommendations align with the World Health Organisation (WHO’s) global strategy for Digital Health [71].

Moreover, as recommended by Neal *et al*. [72], period app users also prefer to have privacy statements in plain and easy-to-understand language. It should also have illustrations and tables to indicate the data that are collected and how they are processed by the company or third parties if any, to help users be aware and make better choices [73].

## LIMITATIONS

A large number of participants were young, middle-class, cis women. However, they were diverse in their cultural beliefs, background and country of residence. For ease of communication, participants who could communicate in English were only chosen. Additionally, there is a chance that individuals who were already aware of privacy issues in these apps chose to participate in this study due to self-selection bias. Future research needs to be conducted with diverse participants, to understand other themes in menstrual app usage.

## CONCLUSION

Through this qualitative study, it is evident that although the apps serve their purpose for them, many participants have recorded their discontentment with the quality and performance of these apps in terms of prediction accuracy, unnecessary data collection and poor data protection measures. It is also notable that a lot of women have become vigilant about data security in recent years, especially with period tracking apps.

Therefore, app developers have to broaden their understanding of female reproductive health needs and create apps that satisfy them. Moreover, lawmakers and app developers must join hands to establish and ensure strict adherence to firm regulations that provide adequate data protection. Besides stricter regulations, apps must have distinct privacy statements that explicitly state the data collection and processing protocol in short and simple terms. Further research needs to be conducted to fully understand the views of other users with different demographics.

## Supplementary Material

OODH_supplementary_materials_oqaf011

## Data Availability

Data are available on request.
