# Supplementary material for: Flowing data: women’s views and experiences on privacy and data security when using menstrual cycle tracking apps
Source: Oxf Open Digit Health. 2025 May 17;3:oqaf011. doi: 10.1093/oodh/oqaf011 (PMC12131320; doi:10.1093/oodh/oqaf011)
Supplement: OODH_supplementary_materials_oqaf011 [file oodh_supplementary_materials_oqaf011.docx]

**Title of the paper:** “Flowing data: Women’s views and experiences on privacy and data security when using menstrual cycle tracking apps”

**Corresponding author:**

Sarika Mohan

Lecturer in Health Informatics

Department: Health Data Science

Swansea University Medical School

Swansea University

Swansea SA2 8PP

Wales

United Kingdom

**Corresponding author Email address**: [sarika.mohan@swansea.ac.uk](mailto:sarika.mohan@swansea.ac.uk)

**Mailing address:** Office number 102 First floor,

Data Science building

Swansea University

Singleton Park

Swansea

SA2 8PP

Wales

United Kingdom

**Author 2**

Judy Jenkins

Programme Director in Health Informatics

Department: Health Data Science

Swansea University Medical School

Swansea University

Swansea SA2 8PP

Wales

United Kingdom

**Acknowledgements**: The authors would like to thank the participants for sharing their unbiased views and experiences and for their participation in the study.

**Study Funding:** No funding was acquired for this study.

**Author contributions:**

Sarika Mohan: Conceptualisation, methodology, investigation, formal analysis and writing.

Judy Jenkins: Supervision, validation

## Appendix A: Semi-structured Interview Questions

1. Please state your name
2. Please state your age
3. How long have you been using menstrual cycle tracking apps?
4. Which app do you use to track your menstrual cycle?
   1. Why did you choose this app?
   2. Is it a free or paid app?
5. Why did you start using the app?
   1. How often do you enter data into the tracker?
   2. What are the factors that you track?
6. What do you find as the most useful feature of this app, and why?
   1. Do you identify any drawbacks/ features that need to be improved or removed?
7. What are your thoughts of the information that is collected?
   1. When you downloaded the app, did you go through the privacy statement of the app? Why/ why not?
   2. Are you aware of the data that is collected, and can you please state them?
   3. Please share your thoughts on the privacy laws of the app you use.
   4. Have you ever felt the need to stop using the app due to the amount of data that is being collected?
8. What security measures would you like to be incorporated in the app for you to use it confidently?
   1. Any specific privacy policies that you want the app to include?
9. Do you have any concerns regarding using the period tracking apps and how your country views women’s health?
   1. Can you state the reasons as to why you are comfortable/ uncomfortable with using the period tracking apps in your country?
10. Thank you for all that valuable information, is there anything else you would like to add before we end?

## Appendix B: Important Quotes from participants

Participant-1: *“I want my period products first”* when asked what their thoughts on their country’s views of women’s reproductive health were.

Participant-2: *“Maybe like, I choose which information to store in the cloud”* regarding what security measures they would like to be incorporated in the future.

Participant-5: *“I just want to make sure that my app data won’t be stolen by some hackers”* when asked about why they need stronger privacy laws.

Participant 6: *“Make it accessible to all people. Make it user-friendly! Make it easy to understand; include sex education and what to do when you have your first period and how your cycle changes after teenage years”* regarding design suggestions they have.

Participant 9: *“Period itself is a big taboo topic to talk about in general, especially when in public and men are involved…. So, I don’t feel that they will be open to say about their sexual life”* on why they think women don’t feel comfortable entering sexual activity in the app. They also said *“add Indian food items as options and not just as a food habit”* regarding design suggestions.

Participant 14: *“you never know where information is going”* about why they felt uncomfortable and stopped using the app.

Participant 22: *“the doctor told me you’re supposed to know, it is your duty as a woman.. I’m not interested, so don’t judge me because I don’t know it”* on why they chose to record their cycle on an app and that they now have a better relationship with their doctor.

Participant 23: *“I was pressured to keep scrolling and scrolling, to something that was not ending, but I felt like it is not enough to protect my data”* on their views of the app’s privacy laws.

**Table 1 App usage and country of residence**

| **Menstrual app used and Country of Residence** | **Count** |
| --- | --- |
| ***Apple Health app*** | ***6*** |
| Canada | 1 |
| India | 1 |
| Jordan | 1 |
| Malaysia | 1 |
| United Kingdom | 2 |
| ***Clue*** | ***7*** |
| India | 3 |
| Jordan | 1 |
| Singapore | 1 |
| United Kingdom | 2 |
| ***Flo*** | ***6*** |
| India | 3 |
| Nigeria | 1 |
| United Kingdom | 1 |
| United States of America | 1 |
| ***My Calendar*** | ***3*** |
| Malaysia | 1 |
| Nigeria | 1 |
| United Kingdom | 1 |
| ***My Period*** | ***2*** |
| Germany | 1 |
| India | 1 |
| ***Period Calendar*** | ***1*** |
| China | 1 |

**Table 2 Age and Education level**

| **Education Level** | **18-21 years** | **22-25 years** | **26-30 years** | **31- 40 years** |
| --- | --- | --- | --- | --- |
| Bachelor’s | 4 | 6 | 4 | 0 |
| Master’s | 0 | 6 | 3 | 2 |
| Total | 4 | 12 | 7 | 2 |
